# A randomised crossover trial of tezacaftor-ivacaftor for gut dysfunction in cystic fibrosis with magnetic resonance imaging (MRI) outcomes.

**DOI:** 10.3310/nihropenres.13510.1

**Published:** 2023-11-27

**Authors:** Christabella Ng, Neele S Dellschaft, Caroline Hoad, Luca Marciani, Robin Spiller, Colin Crooks, Trevor Hill, Alex Menys, Jochen G Mainz, Helen Barr, Penny A. Gowland, Giles Major, Alan R Smyth

**Affiliations:** 1 NIHR Nottingham Biomedical Research Centre, University of Nottingham and Nottingham University Hospitals NHS Trust, Nottingham, UK; 2 Lifespan and Population Health, School of Medicine, University of Nottingham, Nottingham, England, UK; 3 Sir Peter Mansfield Imaging Centre, School of Physics and Astronomy, University of Nottingham, Nottingham, England, UK; 4 Nottingham Digestive Diseases Centre, School of Medicine, University of Nottingham, Nottingham, England, UK; 5 Centre for Medical Imaging, Division of Medicine,, University College London, London, England, UK; 6 Cystic Fibrosis Centre, Brandenburg Medical School, Brandenburg an der Havel, Germany; 7 Wolfson Adult Cystic Fibrosis Unit, Nottingham University Hospitals NHS Trust, Nottingham, UK; 8 Nestlé Institute of Health Sciences, Société des Produits Nestlé, Lausanne, Switzerland; 9 School of medicine Dentistry & Biomedical Sciences, Queens University, Belfast, UK

**Keywords:** cystic fibrosis, gastrointestinal symptoms, CFTR modulators, magnetic resonance imaging.

## Abstract

**Background:**

People with cystic fibrosis (CF) can experience recurrent chest infections, pancreatic exocrine insufficiency and gastrointestinal symptoms. New cystic fibrosis transmembrane conductance regulator (CFTR) modulator drugs improve lung function but gastrointestinal effects are unclear. We aimed to see if a CFTR modulator (tezacaftor-ivacaftor,TEZ/IVA) improves gastrointestinal outcomes in CF.

**Methods:**

We conducted a randomised, double-blind, placebo-controlled, two-period crossover trial (2019-2020) at Nottingham University Hospitals. The effects of TEZ/IVA on gut physiology were measured using MRI. Participants were randomly assigned to treatment sequences AB or BA (A:TEZ/IVA, B:placebo, each 28 days), with a 28-day washout period. Participants had serial MRI scans at baseline and after 19-23 days of each treatment. Due to the COVID-19 pandemic, a protocol amendment allowed for observer-blind comparisons prior to and during TEZ/IVA. In such cases, participants were not blind to the treatment but researchers remained blind. The primary outcome was oro-caecal transit time (OCTT). Secondary outcomes included MRI metrics, symptoms and stool biomarkers.

**Results:**

We randomised 13 participants. Before the COVID-19 pandemic 8 participants completed the full protocol and 1 dropped out. The remaining 4 participants followed the amended protocol. There were no significant differences between placebo and TEZ/IVA for OCTT (TEZ/IVA >360minutes [225,>360] vs. placebo 330minutes [285,>360], p=0.8) or secondary outcomes. There were no adverse events.

**Conclusions:**

Our data contribute to a research gap in the extra-pulmonary effects of CFTR modulators. We found no effect after TEZ/IVA on MRI metrics of gut function, GI symptoms or stool calprotectin. Effects might be detectable with larger studies, longer treatment or more effective CFTR modulators.

**ClinicalTrials.gov registration:**

NCT04006873 (02/07/2019)

## Introduction

Cystic fibrosis (CF) is the most common, life- limiting, autosomal recessive disorder in populations of North European ancestry ^1^. CF is caused by mutations in the gene coding for the cystic fibrosis transmembrane conductance regulator (CFTR): an epithelial chloride channel. Defective CFTR leads to thick, sticky secretions affecting multiple organs and clinical manifestations include lung infections, pancreatic exocrine insufficiency and gastrointestinal (GI) symptoms. Relieving gut symptoms in CF is a priority ^2^, since the majority of people with CF experience gut symptoms ^3^ and two thirds miss school or work as a result of gut symptoms ^4^.

CFTR modulator therapies, for specific CF mutations, aim to correct the defective CFTR protein. The dual CFTR modulator therapy tezacaftor-ivacaftor (TEZ/IVA) is indicated for patients homozygous for the commonest gene mutation causing CF (p.Phe508del) and for those with one copy of p.Phe508del combined with a residual function CFTR mutation.

Over the last decade, the pivotal phase 3 clinical trials for CFTR modulator therapies have focussed on respiratory outcomes, with GI effects reported as adverse effects ^5, 6^. More recent CFTR modulator studies have focussed on nutrition ^7^ and pancreatic exocrine function ^8, 9^, and emerging data on gut symptoms ^10^. Attempts to measure GI function have been limited although some studies have reported the effect of modulators on markers of gut pH ^11^, inflammation and microbiome ^12^.

The introduction of CFTR modulator therapies for a large proportion of people with CF will mean it will be increasingly difficult to compare new therapies with placebo. In this study, we used magnetic resonance imaging (MRI) ^13^ to evaluate the GI effects of a dual combination CFTR modulator in CF and studied the relationship of MRI findings to GI symptoms.

### Aims and objectives

We aimed to evaluate the GI effects of a dual CFTR combination therapy (tezacaftor/ivacaftor; TEZ/IVA) compared to placebo in people with CF, using MRI and symptom questionnaires. We also assessed its effect on stool biomarkers of pancreatic function and intestinal inflammation.

## Methods

### Patient and Public Involvement

The study is based on feedback from previous Patient and Public Involvement, most notably the recent James Lind Priority Setting Partnership for Cystic Fibrosis and the GIFT-CF1 study. The methodology applied has previously been developed with the Patient Advisory Group of the Nottingham Digestive Diseases Biomedical Research Centre. The results of this study will be discussed with patient groups in order to plan future work.

### Study design

We conducted a double-blind, placebo-controlled, randomised crossover trial. Ethical approval was granted by West Midlands – South Birmingham Research Ethics Committee on 28 ^th^ May 2019 (19/WM/0130) and the protocol was prospectively registered (ClinicalTrials.gov; NCT04006873) on 2 ^nd^ July 2019). This article is reported in line with CONSORT ^14^. Dual combination TEZ/IVA was the CFTR modulator chosen for this study because, at the time of study commencement, triple combination therapy was not available in the UK.

### Study population

People with CF aged from 12 to 40 years, homozygous for p.Phe508del and eligible for TEZ/IVA, were invited to participate. Participants were recruited from the local tertiary CF centre, Nottingham University Hospitals NHS Trust, UK, based upon pre-specified inclusion and exclusion criteria ( Table 1). Written informed consent was obtained.

**Table 1.** Summary of inclusion and exclusion criteria.

Inclusion criteria	Exclusion criteria
• Capacity to consent, or understand the requirements of the study where parental consent is needed • Confirmed diagnosis of CF (homozygous p.Phe508del)	• Currently taking a CFTR modulator drug • Contra-indication to use of TEZ/IVA • FEV _1_ <40% predicted using the Global Lung Initiative criteria ^15^ • Contra-indication to MRI scanning, such as embedded metal or pacemaker • Pregnancy • Unable to stop medications directly prescribed to alter bowel habit such as laxatives or anti-diarrhoeals on the study day • Previous resection of any part of the GI tract apart from appendicectomy or cholecystectomy. Surgical relief of meconium ileus or DIOS will be permitted unless clinical records show excision of intestine >20 cm in length • Intestinal stoma • Diagnosis of inflammatory bowel disease or coeliac disease confirmed by biopsy • GI malignancy • Unable to comply with dietary restrictions required for the study

### Study procedures

Before randomisation, participants underwent a series of MRI scans on a single day ^13^. MRI scans from our previous study ^13^ were used for the baseline data if participants had enrolled to both studies. At the time this trial commenced, TEZ/IVA was licensed in the UK but not reimbursed, so was not prescribed in routine clinical care. Nottingham University Hospital trials pharmacy performed secure web based simple randomisation, using an online computer program ( www.randomization.com) which created a computer-generated list and ensured allocation concealment. Participants were randomly assigned to treatment sequences AB or BA (A: TEZ/IVA, B: placebo), with a 28-day washout period in between. The investigators and participants were blind to treatment allocation. TEZ/IVA and placebo were visually identical.

**Figure 1.**
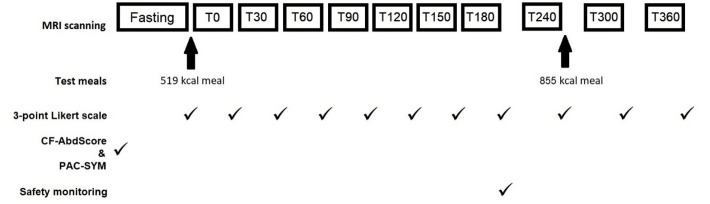
Diagram to show study procedure timings. MRI scans performed at fasting, at half hourly intervals until 180 minutes after the first meal (T180), then hourly intervals until T360. Standardised test meals were given after the fasting scan and T240 scan. CFAbd-Score and PAC-SYM questionnaires were completed prior to fasting scan. A 3-point Likert scale was completed after each scan. Safety monitoring was undertaken after the T180 scan.

Outcomes were measured between day 19 and 23 of treatment, according to participant and scanner availability. The study visit consisted of a series of 11 MRI scans and GI symptom assessment as previously described ^13, 16^ in fasted and postprandial states ( Figure 1). All participants fasted for at least 12 hours before attending, other than water for essential medicines.

Each participant was given the same rice pudding meal (519 kcal, fat 19g, carbohydrate 77g) ^13^, immediately after the fasting scan. A second meal (855 kcal, fat 41g, carbohydrate 91g) was eaten at approximately 265 minutes after the first meal and consisted of 375g macaroni cheese (Sainsbury’s, UK); 100g strawberry cheesecake (Rhokett, UK); and 240mL water. No other food and drink were permitted during the visit. Participants took their prescribed amount of pancreatic enzyme supplements with meals. No laxatives or anti-diarrhoeals were permitted during the visit.

Participants provided a stool sample at each study visit for faecal elastase and calprotectin analysis. Stool samples timings were restricted to 12 hours preceding and 24 hours post study visit. Blood pressure measurement, serum liver and renal function tests were performed for safety monitoring ^17^ at each visit. Tablet blister packs were retrieved to assess participant adherence to the study medication. Drug levels were not measured.

### Scanning protocol

A 3-Tesla Philips Ingenia MRI scanner (Philips Healthcare, Best, The Netherlands) was used. Participants were positioned supine with a DS anterior coil over the abdomen to capture MR images ^13^. We evaluated gut function and transit using previously published MRI techniques ^13, 16, 18^. Scan times were defined relative to ingestion of the first meal (i.e. T0 was the MRI scan taken at 0 minutes after the first meal was consumed).

### Mitigations for COVID-19 pandemic disruptions

Study enrolment commenced in September 2019. In March 2020, in line with UK government rules to restrict the transmission of SARS-CoV2 virus, our centre suspended all research involving direct contact with participants (apart from COVID-19 studies). Our trial was paused between mid-March and August 2020. During this period, reimbursement of TEZ/IVA was introduced in the UK and 4 participants commenced TEZ/IVA as part of their routine CF treatment. It was unethical to request their return to the study protocol involving placebo treatment. These 4 participants therefore completed observer-blind study visits when research resumed. Observer-blind visits were single-blind, where the observers conducting the analysis were blind to treatment status.

### Data analysis

**Figure 2.**
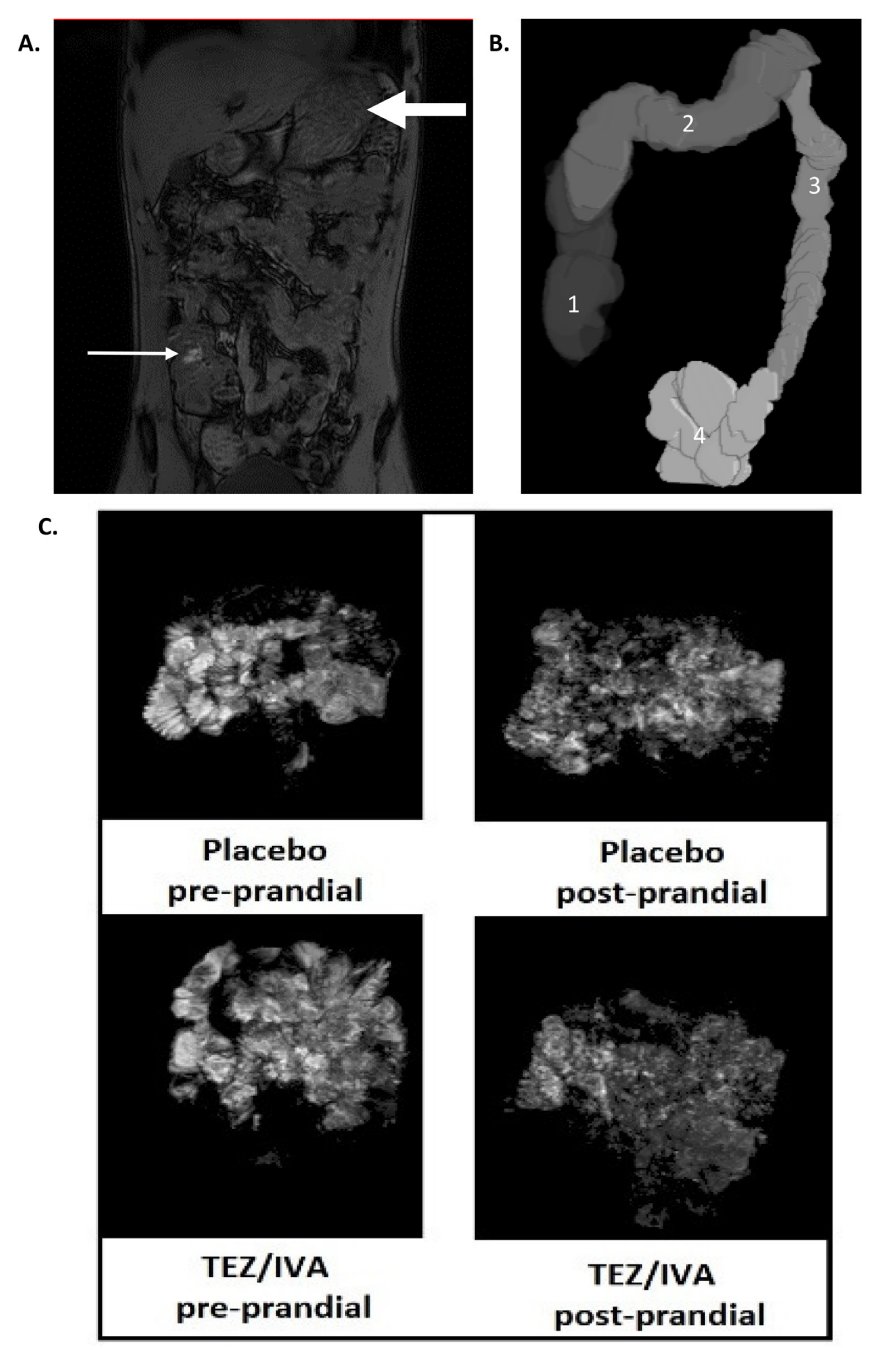
Representative MRI images of ( **A**) dual echo for orocaecal transit time (OCTT) and colonic volumes (small arrow: head of the meal in the caecum, large arrow: stomach; ( **B**) 3D render of colonic volumes (1: ascending colon, 2: transverse colon, 3: descending colon, 4: recto-sigmoid colon); ( **C**) small bowel water content (SBWC) analysis pre and post prandial for placebo and TEZ/IVA. The greyscale demonstrates the signal intensity of small bowel water and its threshold is compared to cerebrospinal fluid of individual participants.

MRI analysis was performed using Medical Image Processing, Analysis and Visualisation ^19^ (MIPAV, NIH, Bethesda, MD, USA) and GIQuant ^®^ (Motilent, London, UK). We also used some, in-house software written in MATLAB ^®^ (The MathWorks Inc., Natick, MA, USA). Example images are shown in Figure 2.

### Outcome measures

The primary outcome was oro-caecal transit time (OCTT), defined as the scan time when the meal is first detected in the caecum.

The secondary outcomes included:

- corrected colonic volumes, defined as the sum of the ascending, transverse, descending and recto-sigmoid colon divided by body surface area ^13^. Mosteller’s formula ^20^ was used to calculate participant’s body surface area.
- corrected small bowel water content (SBWC), defined as SBWC divided by body surface area ^13^. Small bowel water is the volume of free water within the small bowel;
- postprandial change in corrected SBWC after the second meal (delta SBW), defined as the difference in corrected SBWC between T240 and T300 ^13^;
- gastric half-emptying times, defined as the time taken for half the initial gastric contents to empty into the duodenum ^21^; and
- GI symptoms, using the PAC-SYM ^22^ (patient assessment of constipation symptoms) questionnaire, CFAbd-Score ^3^, and a 3-point Likert scale to record abdominal pain, bloating and flatulence concurrently with each MRI scan (for an example see Table 2).

Our exploratory outcomes were T _1_ relaxation time of chyme in the ascending colon as a measure of water content; terminal ileum motility; faecal elastase; and faecal calprotectin. We have published the results of faecal microbiome analysis in a second publication ^23^.

**Table 2.** Example of the Likert scale used at each MRI timepoint for flatulence, bloating and abdominal pain.

Time point:	FASTING	Time is now:	H	H	:	M	M
check **ONE** box that **best** describes how bad each symptom is **right now:** **0 = not at all** **1 = mild** (distinct but negligible) **2 = moderate** (annoying) **3 = severe** (disabling)							
**Flatulence (Wind):**	**0**	**0.5**	**1**	**1.5**	**2**	**2.5**	**3**
**Bloating:**	**0**	**0.5**	**1**	**1.5**	**2**	**2.5**	**3**
**Abdominal Pain:**	**0**	**0.5**	**1**	**1.5**	**2**	**2.5**	**3**

### Statistical analysis

This is the first study to evaluate within-individual differences in MRI metrics for people with CF. Little is known about the minimum clinically important difference for OCTT caused by CFTR modulators. There are insufficient data to provide an informative power calculation and therefore, a sample size of 12 was chosen ^24^. In total, 15 individuals were recruited to allow for attrition.

We used within-participant comparisons for the crossover analyses to compare TEZ/IVA with placebo. Period and carry-over effects were minimised with the 28-day washout period. Kaplan-Maier and log-rank tests were used to calculate statistical significance for OCTT. Non-parametric tests were planned for secondary and exploratory outcomes, with data presented as median (inter-quartile range; IQR). All statistical tests were conducted using RStudio Version 4.1.2 (RStudio, PBC, Boston, MA, USA). A p-value of <0.05 was considered statistically significant.

In view of COVID-19 disruptions, we completed a primary per protocol analysis for all participants who were blind to treatment. A secondary post-hoc intention-to-treat analysis compared all enrolled participants who attended study visits on and off TEZ/IVA.

## Results

### Study progress

**Figure 3.**
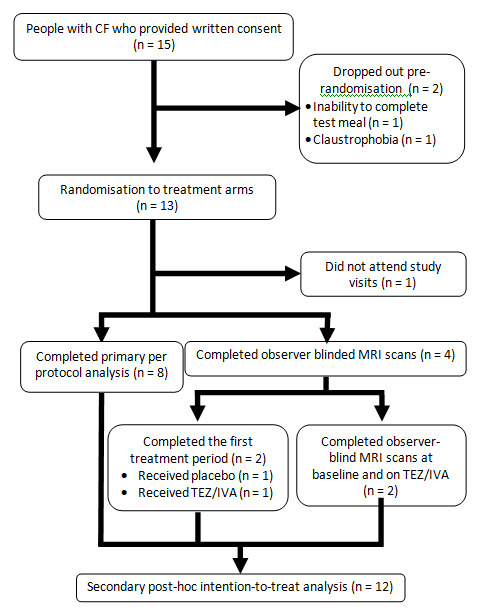
Consort diagram to show participant flow.

All procedures took place between September 2019 and October 2020. In total, 15 people with CF were enrolled ( Figure 3), of whom 7 had participated in the previous GIFT-CF 1 study. Two participants dropped out at the time of the baseline scan as they were unable to complete the first meal or experienced claustrophobia in the scanner. One participant did not attend the post-randomisation study visits. Of the remaining 12 participants, 8 completed both treatment periods and all study visits per protocol and 4 underwent observer-blind study visits on and off TEZ/IVA.

Participants’ baseline characteristics are summarised in Table 3. Baseline scan data were obtained during the GIFT-CF1 study ^13^ for 7 participants. The median baseline values were as follows: age 20 years; OCTT 330 minutes; corrected SBWC AUC 42 L∙min/m ^2^; Delta SBW 19 mL/m ^2^; corrected fasting colon volume 291 mL/m ^2^; CFAbd-Score 16.2; and PAC-SYM 0.38. One participant had a history of surgically-managed neonatal meconium ileus. There was no documentation of the length of small bowel removed in this case and therefore the participant was included. Another participant had a history of medically-managed DIOS.

**Table 3.** Participant demographics at baseline. ^†^Baseline scan data for 7 participants were from the previous GIFT-CF1 study (ClinicalTrials.gov NCT03566550).

*Characteristic, median (Q25, Q75)*	N = 12
Gender, M:F	8 : 4
Age, years	20 (17, 26)
Forced Expiratory Volume in 1 second (FEV _1_), %	80 (59, 100)
Height, cm	167 (161, 176)
Weight, kg	59 (55,65)
*Baseline ^†^ visit, median (Q25, Q75)*	N = 12
OCTT, mins	330 (300, >360)
Corrected SBWC AUC, L∙min/m ^2^	64 (53, 75)
Delta SBW, mL/m ^2^	19 (11, 60)
Corrected Fasting colon volume, mL/m ^2^	291 (190, 437)
CFAbd-Score	16.2 (13.8, 21.7)
PAC-SYM	0.38 (0.25, 0.5)

There was more than 85% adherence for both treatment periods in 7 out of the 8 (87.5%) participants who completed the study double-blind. One participant had 57% adherence in the TEZ/IVA arm only.

Five participants received antimicrobial therapy whilst enrolled in the study: 1 received oral antibiotics pre-randomisation, 2 received oral antibiotics during the first period, 1 received oral antibiotics during the washout period and 1 commenced itraconazole during study pause period.

Of 4 participants who underwent observer-blind study visits, 2 were randomised pre-pandemic (one had placebo and one had TEZ/IVA in the first treatment period). Neither participant started the second treatment period due to COVID-19 disruptions. Both underwent observer-blind study visits whilst taking TEZ/IVA. Therefore, the within-participant comparison was between placebo and TEZ/IVA, and baseline and TEZ/IVA respectively. There were 2 participants who underwent observer-blind visits at baseline and on TEZ/IVA (prescribed as part of routine clinical care). All outcomes reported are primary per protocol analysis (n=8) unless otherwise specified ( Table 4). There were no adverse events highlighted from the safety monitoring.

**Table 4.** Summary of primary, secondary and exploratory outcomes. Statistical tests used: Log-rank used for OCTT, Wilcoxon signed-ranks test for all other metrics. For terminal ileum motility: ^†^ n = 10, ^‡^ n = 8, ^§^ n = 7, ^¶^ n = 6.

	Primary per protocol analysis (n = 8)			Secondary post-hoc analysis (n = 12)		
Outcome Median (Q25, Q75)	TEZ/IVA	Placebo	p-value	TEZ/IVA	No TEZ/IVA	p-value
OCTT *(mins)*	>360 (225, >360)	330 (285, >360)	0.80	> 360 (225, >360)	360 (300, >360)	> 0.99
Gastric half-emptying time *(mins)*	96 (70, 105)	95 (70, 110)	0.57	92 (70, 105)	86 (67, 101)	0.48
Delta SBW *(mL/m ^2^)*	35 (-67, 64)	3 (-8, 33)	>0.99	28 (-64, 64)	19 (2, 50)	0.27
Corrected SBWC AUC *(L∙min /m ^2^)*	49 (39, 69)	35 (29, 42)	0.08	45 (36 ,62)	34 (29, 49)	0.12
Corrected colonic volumes AUC *(L∙min /m ^2^)*	179 (156, 232)	167 (144, 211)	0.46	179 (151, 211)	172 (150, 186)	0.73
CFAbd-Score	17.2 (12.1, 27.4)	21.2 (16.9, 27.8)	0.45	12.2 (7.2, 21.8)	18.9 (13.2, 22.9)	0.20
PAC-SYM	0.63 (0.08, 0.88)	0.71 (0.50, 0.90)	0.68	0.25 (0.08, 0.71)	0.67 (0.13, 0.83)	0.41
Total Likert scores	3.5 (0, 9.63)	2.75 (0.88, 9.50)	0.50	3.5 (0, 5.25)	2.75 (0.38, 8.75)	0.64
Terminal ileum motility *(arbitrary* * units, au) Fasting*	0.15 ^§^ (0.13, 0.23)	0.17 ^§^ (0.11, 0.21)	0.94	0.15 ^†^ (0.11, 0.22)	0.15 ^†^ (0.10, 0.19)	0.92
*Postprandial T0*	0.20 ^¶^ (0.16, 0.21)	0.23 ^¶^ (0.14, 0.26)	>0.99	0.20 ^‡^ (0.15, 0.24)	0.23 ^‡^ (0.19, 0.27)	0.74
*Postprandial T300*	0.16 ^‡^ (0.13, 0.19)	0.19 ^‡^ (0.18, 0.22)	0.38	0.16 ^†^ (0.14, 0.21)	0.19 ^†^ (0.15, 0.21)	0.21
Fasting T _1_AC *(secs)*	0.66 (0.61, 0.82)	0.49 (0.41, 0.97)	0.74	0.65 (0.51, 0.80)	0.55 (0.43, 0.95)	0.97
Faecal elastase *(μg/g)*	4.9 (2.0, 6.5)	2.5 (1.2, 7.7)	0.84	1.9 (<1.0, 5.6)	<1.0 (<1.0, 7.0)	0.42
Faecal calprotectin *(μg/g)*	13.7 (5.2, 24.9)	16.4 (8.0, 23.6)	0.64	13.7 (5.2, 25.8)	12.6 (8.0, 21.5)	0.73

### Primary outcome

**Figure 4.**
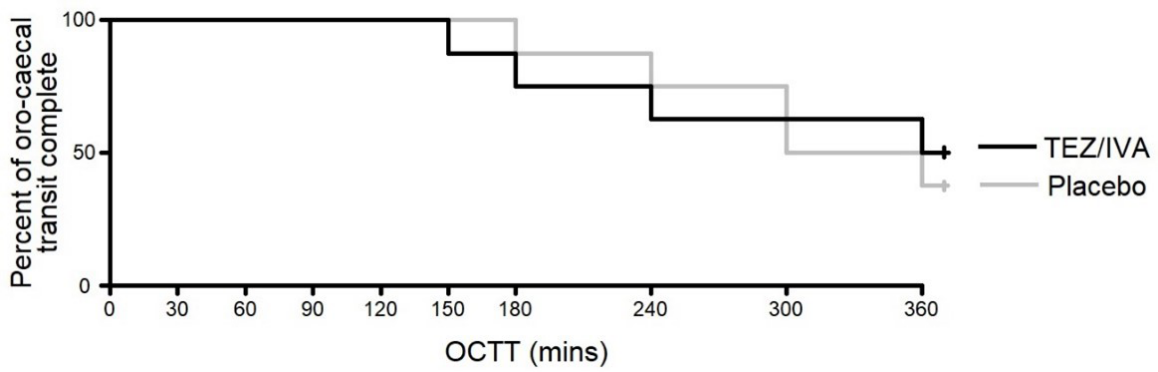
Graph to show the time taken for a meal to reach the caecum in the primary analysis (n = 8). Number censored TEZ/IVA = 4, placebo = 3.

There was no significant difference between the treatment groups for OCTT (TEZ/IVA >360 minutes [225, >360] vs. placebo 330 minutes [285, >360], p = 0.8, Figure 4). An OCTT over 360 minutes was not quantified as the last MRI scan was at 360 minutes. For 2 participants, the meal had not reached the caecum by 360 minutes in either treatment period. This was also true for a further participant on placebo only and 2 participants on TEZ/IVA only.

### Secondary outcomes

There were no significant differences between the two groups for the secondary and exploratory outcomes.

***Corrected SBWC and delta SBW***

**Figure 5.**
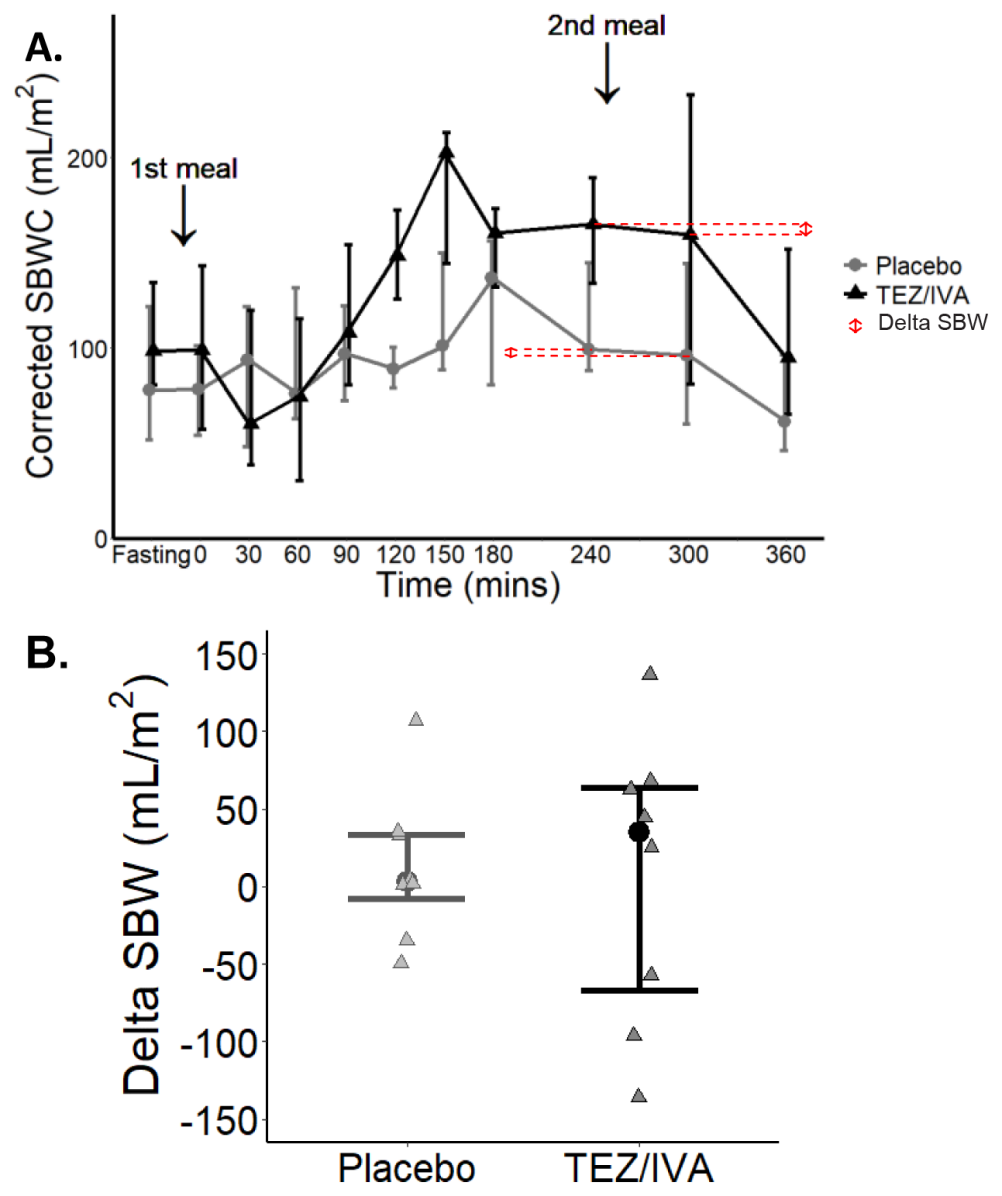
Graphs for primary analysis (n = 8) of corrected SBWC and delta SBW. ( **A**) Median (▴) and IQR (whiskers) changes in corrected SBWC during a study day. ↕ shows the delta SBW determined from the difference in corrected SBWC at 240 and 300 minutes for the averaged dataset. ( **B**) Delta SBW for each participant (▴), error bars to show median and IQR.

The corrected SBWC area under the curve (AUC) was not different between the treatment groups (TEZ/IVA (49 L∙min/m ^2^ [39, 69] vs. placebo 35 L∙min /m ^2^ [29, 42], p = 0.08, Figure 5A). There was no difference in delta SBW ( Figure 5B) between the two treatment groups TEZ/IVA 35mL/m ^2^ [-67, 64] vs. placebo 3mL/m ^2^ [-8, 33], p > 0.99).

**Figure 6.**
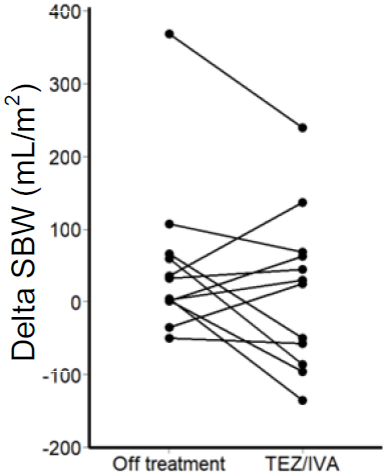
Graph for secondary analysis (n = 12) of corrected delta SBW. Change in corrected delta SBW between off treatment and during TEZ/IVA for each participant.

Of note, there was an outlier participant in the secondary post-hoc analysis whose delta SBWC was more than four times that of the upper quartile in the intention-to-treat analysis (TEZ/IVA 286mL/m ^2^, off treatment 368mL/m ^2^, Figure 6). Larger delta SBW indicates a larger volume of small bowel water has passed through the ileo-caecal valve over 1 hour, following the stimulus of a standardised meal.

***Corrected colonic volumes***

**Figure 7.**
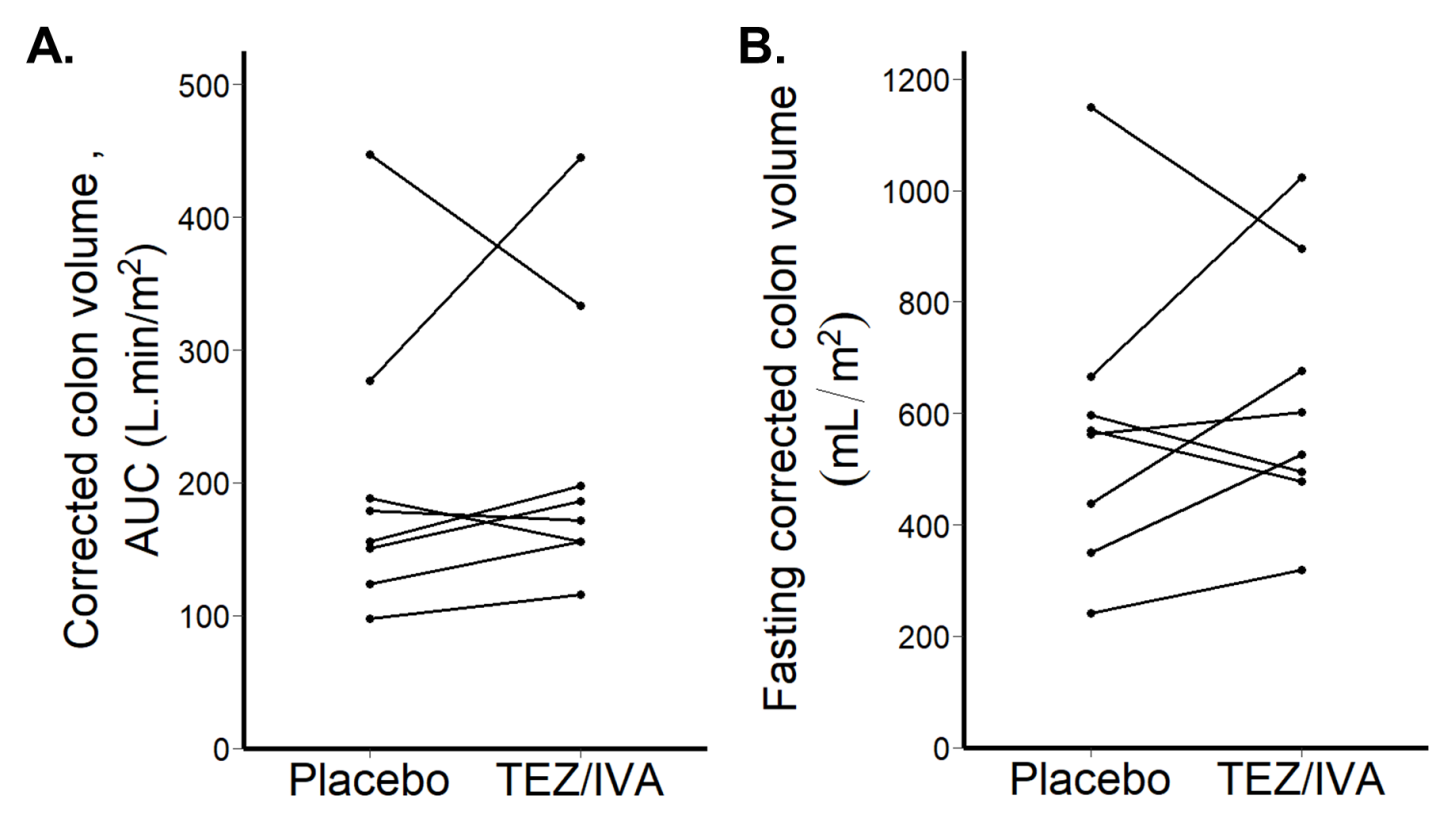
Graphs for primary analysis (n = 8) of corrected colon volumes. ( **A**) Corrected colon volume area under the curve (AUC) after 21 days treatment. ( **B**) Fasting corrected colon volume after 21 days treatment.

Corrected colonic volumes AUC were not different between the groups (TEZ/IVA 179 L∙min/m ^2^ [156, 232] vs placebo 167 L∙min/m ^2^ [144, 211], p = 0.46, Figure 7A).

The corrected fasting colonic volumes were not different between the groups (TEZ/IVA 565mL/m ^2^ [490, 732] vs. placebo 566mL/m ^2^ [416, 614], p = 0.64, Figure 7B).

***Gastric half-emptying times***

Gastric half-emptying times were not different (TEZ/IVA 96 minutes [70, 105] vs. placebo 96 minutes [70, 110], p = 0.57)

***GI symptoms***

**Figure 8.**
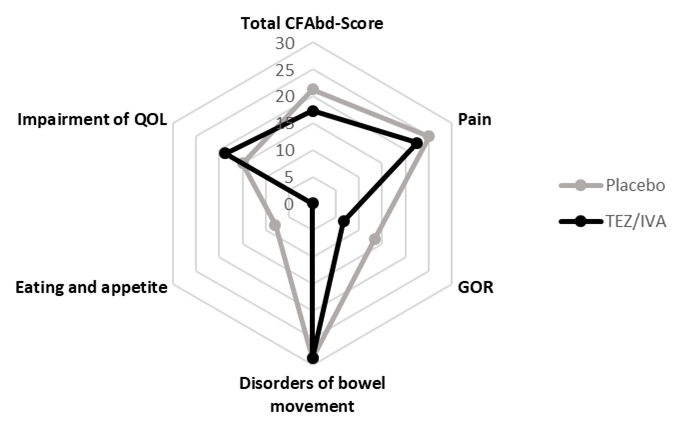
Diagram to show median scores for the total CFAbd-Score as well as for each of the five domains of the CFAbd-Score in the primary analysis (n = 8). GOR: gastro-oesophageal reflux, QOL: quality of life. A higher domain or total score reflects a higher symptom severity.

There was no difference between treatment periods in the GI symptoms assessed by PAC-SYM (TEZ/IVA 0.63 [0.08, 0.88] vs. placebo 0.71 [0.13, 0.83], p = 0.68) or CFAbd-Score questionnaires (TEZ/IVA 17.2 [12.1, 27.4] vs. placebo 21.2 [16.9, 27.8], p = 0.45, Figure 8). The highest scoring domain within the CFAbd-Score was disorders of bowel movement and the lowest scoring domain was eating and appetite ( Figure 8). During the visit, the total Likert scores did not differ between the groups (TEZ/IVA 3.5 [0, 9.63] vs placebo 2.75 [0.88, 9.5], p = 0.50). Individual domains within the Likert score (flatulence; bloating; abdominal pain) remained low, with minimal increases postprandially during TEZ/IVA.

### Exploratory outcomes

***MRI analysis***

The terminal ileum motility scores ( Table 4) were low throughout the study day on placebo and after TEZ/IVA. In inflammatory bowel disease, a low motility score has been shown to be associated with active ileal inflammation ^18^. The fasting and postprandial terminal ileum motility scores were not different when on placebo or TEZ/IVA. The terminal ileum was not visualised in 1 participant at fasting for both TEZ/IVA and placebo or in 2 participants at T0, and therefore they were excluded from analysis. The T _1_ analysis of the ascending colon at fasting was low in both treatment groups but not different between placebo and TEZ/IVA ( Table 4).

***Stool markers***

The median faecal elastase and calprotectin were low in both treatment groups.

## Discussion

Our study is the first randomised placebo-controlled trial to evaluate the GI effects of a CFTR modulator using MRI. These data provide valuable insights in both objective and subjective measurements. Our study showed no significant difference after TEZ/IVA in GI function, transit, symptoms or in stool markers of inflammation or pancreatic function. A longer treatment period, larger sample size or more potent modulator may be needed to show a difference, if a true difference is present. Newer triple combination CFTR modulator therapies have proven to be more potent in improving lung function and nutritional status ^25^ than single or dual combination CFTR modulators.

We observed a prolonged OCTT, in study participants, similar to other studies ^13, 26, 27^, despite a period of TEZ/IVA. Wireless capsule studies showed no change in small bowel transit before and after ivacaftor ^11^, although there was an increase in small intestinal pH. A CFTR modulator may increase intestinal pH and influence the overall balance of GI secretions. However, any changes in pancreatic bicarbonate secretion with ivacaftor may not be enough to affect the overall viscosity of small bowel content and thus transit times in CF.

Compared to previous MRI studies using healthy volunteers, our new CF data show a higher SBWC ^28^ and a lower water content of the ascending colon chyme ^29^. Whilst we do not see any changes in corrected SBWC after TEZ/IVA, we did observe the fluctuations in pre and postprandial small bowel water ( Figure 5A) which may reflect the secretion and absorption actions within the healthy small bowel ^30^. The relative contribution of pancreatic secretion cannot be determined from these data and would require dedicated pancreatic imaging ^31^.

A low delta SBW indicates less small bowel water passing through the ileo-caecal valve in response to a meal and could reflect a partial obstruction of the terminal ileum in CF ^13^. A negative delta SBW suggests small bowel water build up. The post-hoc analysis delta SBW outlier was the participant with a history of neonatal meconium ileus and may reflect the effects of surgery. Such a high delta SBW value (indicating an increased volume passing through the ileo-caecal valve) suggests that a shorter small bowel may lead to faster transit and there may be little or no obstruction at the ileo-caecal valve.

We intentionally focused on the terminal ileum because this is where we believe the underlying pathology exists for CF GI problems. Reduced small intestinal motility using capsule endoscopy ^27^ and MRI ^16^ has been reported in CF compared to controls. Low motility scores correlate with active inflammation seen in inflammatory bowel disease by endoscopy and histopathology ^18^. However direct comparison with our study is difficult since different motility patterns may be induced with different stimuli (bowel preparation vs. high calorie meal). We did not find raised faecal calprotectin levels, which suggests that other factors other than inflammation may explain the cause of low terminal ileum motility and low delta SBW.

Recent studies ^8, 9^ have suggested pancreatic exocrine function recovery with ivacaftor after 24 weeks in younger cohorts. Whilst studies have demonstrated significant improvements in lung function after a month of TEZ/IVA ^6^, this may not be the case for pancreatic function as shown in our study. This suggests that, if pancreatic recovery were possible, it may occur only in the younger population or require a longer treatment period with CFTR modulators or more efficacious CFTR modulators.

We saw no significant difference in GI symptoms after TEZ/IVA. It is likely that a much larger sample size would be required to see an effect on these subjective outcomes ^10^.

Whilst we have not seen any difference in a range of GI measures during TEZ/IVA compared to placebo, other published data suggest there may be value in evaluating GI function over longer periods and with triple combination CFTR modulators. *In vitro* studies, using intestinal organoids from individuals with CF, are supportive of the return of intestinal CFTR function with both TEZ/IVA and triple combination CFTR therapy ^32^.

### Strengths and weaknesses

Our study contributes to the growing evidence for GI pathophysiology in CF using non-invasive techniques. We present data in keeping with our previous findings ^13^ and with wireless capsule data ^11^.

Other studies have made attempts to evaluate GI function, but there are few that are placebo-controlled. Approximately 80% of people with CF are eligible for a CFTR modulator at present ^33^. Trials which aim to compare new modulators with placebo will be difficult as it may be considered unethical for patients to stop their modulator therapy to participate in a placebo-controlled trial.

We acknowledge our study’s limitations, including the 6 hour MRI protocol which could be burdensome and impractical for routine clinical use. We plan to develop shorter, simplified MRI protocols and the metric, delta SBW, shows promise in this regard.

Other limitations include the relatively short treatment periods, the use of TEZ/IVA; a less potent CFTR modulator than elexacaftor/tezacaftor/ivacaftor, and the true TEZ/IVA levels were not measured. Wireless capsule data ^11^ suggest small intestinal pH changes after 1 month on ivacaftor and therefore we hypothesised that changes in MRI parameters may also be seen. Although we have not shown a change in GI function with TEZ/IVA, this is the first randomised controlled trial with objective GI outcomes.

## Conclusions

We have successfully conducted a placebo controlled, cross-over trial to compare TEZ/IVA with placebo and report no change in GI function and symptoms after receiving approximately 21 days of TEZ/IVA. Our data contribute to the existing data on GI MRI outcomes in CF and will inform future sample size calculations. Future studies including longitudinal assessment of triple combination CFTR modulators are indicated to evaluate their GI effects in the long term and aid our understanding of GI function in CF. Future studies should guide treatments of GI symptoms and complications in the presence of triple combination modulators.

## Data Availability

Due to the small sample size, the dataset cannot be effectively deidentified. As required by the research ethics committee, data, with personal identifiers removed can only be made available upon receipt of a reasonable request to the corresponding author ( alan.smyth@nottingham.ac.uk). MRI scans carry the risk of identifying the participant and will not be provided. As the in-house code written for the analysis is specific to this study, interested readers can apply for access from the corresponding author to allow discussion of how it should be used ( alan.smyth@nottingham.ac.uk). Zenodo: Complete CONSORT checklist for ‘A randomised crossover trial of tezacaftor-ivacaftor for gut dysfunction in cystic fibrosis with magnetic resonance imaging (MRI) outcomes.’ https://doi.org/10.5281/zenodo.10118631 ^14^. Data are available under the terms of the Creative Commons Zero "No rights reserved" data waiver (CC0 1.0 Public domain dedication).
