# Supplementary material for: A randomised crossover trial of tezacaftor-ivacaftor for gut dysfunction in cystic fibrosis with magnetic resonance imaging (MRI) outcomes: a pilot study
Source: NIHR Open Res. 2024 Mar 19;3:65. Originally published 2023 Nov 27. [Version 2] doi: 10.3310/nihropenres.13510.2 (PMC11320032; doi:10.3310/nihropenres.13510.2)
Supplement: Peer review history

# Reviewer report: Reviewer response for version 2

**DOI:** 10.3310/nihropenres.14726.r31412

**Published:** 2024-04-29

**Authors:** Dipesh Vasant

**Affiliations:** 1 Manchester University NHS FoundationTrust, Manchester, UK

This is a novel study measuring the effects of initiating a CFTR gene modulator therapy of gut symptoms and gut transit and motility using MRI methodology. 

 The study has a number of limitations which have appropriately been addressed by the authors.

 The main limitations are the lower than planned recruitment which means that this is not a definitively negative study, the lack of longer term follow up, and the applicability of the MRI technology to clinical practice. Nonetheless, the study design is excellent, robust, and is important mechanistic work. 

 I have no major concerns with the paper.

 Minor comments:

 1. In the exploratory outcomes section of the main text the authors have stated stool tests - with low faecal calprotectin and low elastase results, please could the authors cite the appropriate table where this data is included in the main text.

 2. It is not clear whether patients were allowed to take/remain on potential confounding medications such as laxatives, prokinetics, anti-motility drugs which could have affected results.

 3. At day 19-23 where follow-up was performed, is there any literature to suggest that even the non-GI symptoms and pulmonary function, weight can improve within such a short time frame? Were any non-GI effects of the gene modulators within the study duration documented.

Is the work clearly and accurately presented and does it cite the current literature?

Yes

If applicable, is the statistical analysis and its interpretation appropriate?

Yes

Are all the source data underlying the results available to ensure full reproducibility?

Yes

Is the study design appropriate and is the work technically sound?

Yes

Are the conclusions drawn adequately supported by the results?

Yes

Are sufficient details of methods and analysis provided to allow replication by others?

Yes

Reviewer Expertise:

GI motility and gastrointestinal disease in CF.

I confirm that I have read this submission and believe that I have an appropriate level of expertise to confirm that it is of an acceptable scientific standard, however I have significant reservations, as outlined above.

# Reviewer report: Reviewer response for version 2

**DOI:** 10.3310/nihropenres.14726.r31411

**Published:** 2024-04-12

**Authors:** Mette Frahm Olsen

**Affiliations:** 1 University of Copenhagen, Copenhagen, Denmark

Abstract:

 It is unclear why the protocol was amended with observer-blind assessments due to COVID-19. Could you clarify how this was a consequence of the pandemic?

 Methods:

 The study design is described as double-blind here, but some participants were not blinded. You could describe it as outcome assessor/observer blind or explain further.

 What was the reason for the age range of invited patients (12-40 y)?

 The age range is not mentioned as an inclusion criteria in Table 1. Please add.

 Was the randomization blocked?

 Please clarify the timing of study visits. You write that all measurements were done on one day between day 19 and 23 of treatment. This is then repeated for the two periods of the cross-over study, right?

 You write that it was unethical for participants to be blinded to their treatment status after the COVID interruption. I am not sure I understand why is blinding was considered unethical to participants after COVID – but not before?

 Results:

 Please add the two study arms to the CONSORT flow chart (fig 3), i.e. treatment/placebo and placebo/treatment

 Add a brief explanation of the GIFT-CF 1 study (overall aim, methods and perhaps findings)

 Please clarify if higher scores of the various patient-reported outcomes indicate worse or better outcomes.  

 Discussion:

 Please comment on potential impact of the double-blinding vs observer-blinding. Do you consider your outcomes to be prone to bias when the participant is not blinded? I would assume that your primary outcome is objective enough to not be affected by detection bias, so why have you chosen the smaller blinded group for your primary analysis?

Is the work clearly and accurately presented and does it cite the current literature?

Yes

If applicable, is the statistical analysis and its interpretation appropriate?

Yes

Are all the source data underlying the results available to ensure full reproducibility?

No

Is the study design appropriate and is the work technically sound?

Yes

Are the conclusions drawn adequately supported by the results?

Yes

Are sufficient details of methods and analysis provided to allow replication by others?

Partly

Reviewer Expertise:

CF epidemiology, trial methodology

I confirm that I have read this submission and believe that I have an appropriate level of expertise to confirm that it is of an acceptable scientific standard, however I have significant reservations, as outlined above.

# Response

**Published:** 2024-04-12

**Authors:** Alan Smyth

**Affiliations:** Lifespan and population health, University of Nottingham, Nottingham, England, UK

Dear Dr Olsen. Thank you for your interest in our research and for taking the time to review our paper.

**Reviewer's question :**

 Abstract: It is unclear why the protocol was amended with observer-blind assessments due to COVID-19. Could you clarify how this was a consequence of the pandemic?

**Response:** Study enrolment commenced in September 2019. In March 2020, in line with UK government rules to restrict the transmission of SARS-CoV2 virus, our centre suspended all research involving direct contact with participants (apart from COVID-19 studies). Our trial was paused between mid-March and August 2020. During this period, reimbursement of TEZ/IVA was introduced in the UK and 4 participants commenced TEZ/IVA as part of their routine CF treatment. It was unethical to request their return to the study protocol involving placebo treatment. These 4 participants therefore completed observer-blind study visits when research resumed. Observer-blind visits were single-blind, where the observers conducting the analysis were blind to treatment status. These 4 participants had MRI studies pre-treatment and whilst taking TEZ/IVA prescribed by their CF Centre. This is explained in the METHODS section under "Mitigations for COVID-19 pandemic disruptions".

**Reviewer's question :**

 Methods: The study design is described as double-blind here, but some participants were not blinded. You could describe it as outcome assessor/observer blind or explain further.

**Response: **See above. The 4 study participants who had an MRI study on routinely prescribed TEZ/IVA knew what treatment they were taking but the research staff, who scanned the participant and interpreted the scans, were blind to whether or not the participant was taking TEZ/IVA (observer blind). This is explained in the METHODS section under "Mitigations for COVID-19 pandemic disruptions".

**Reviewer's question : **What was the reason for the age range of invited patients (12-40 y)?

**Response: **This is the age group for whom TEZ/IVA was licensed in the UK.

**Reviewer's question : **The age range is not mentioned as an inclusion criteria in Table 1. Please add.

**Response:** People with CF aged from 12 to 40 years, homozygous for p.Phe508del and eligible for TEZ/IVA, were invited to participate. This is explained in the METHODS section under "Study population".

**Reviewer's question : **Was the randomization blocked?

**Response: **No we used simple randomization.

**Reviewer's question : **Please clarify the timing of study visits. You write that all measurements were done on one day between day 19 and 23 of treatment. This is then repeated for the two periods of the cross-over study, right?

**Response: **Correct. We aimed to do the scan on day 21 of treatment but had to have a window of day 19 to 23 of treatment to allow for participants having other commitments on day 21 or the scanner having a previous booking for that day.

**Reviewer's question : **You write that it was unethical for participants to be blinded to their treatment status after the COVID interruption. I am not sure I understand why is blinding was considered unethical to participants after COVID – but not before?

**Response: **We planned a placebo controlled study because TEZ/IVA was licensed in the UK at the time but was not reimbursed and so not routinely prescribed in UK CF Centres. The study was paused, due to the COVID pandemic, from March-August 2020. Reimbursement for TEZ/IVA was introduced by the UK Department of Health and Social Care in mid-2020 and so 4 patients received the drug from their CF Centre during the study pause. We felt it would be unethical to ask these patients to continue on a protocol which required 28 days TEZ/IVA - 28 days washout - 28 days placebo (or the reverse order) as both the washout and placebo periods would deny them modulator treatment. We therefore undertook an observer blind MRI assessment whilst the participant was on TEZ/IVA treatment and compared that to the same participant's baseline MRI (no modulator treatment). 

**Reviewer's question :**

 Results: Please add the two study arms to the CONSORT flow chart (fig 3), i.e. treatment/placebo and placebo/treatment

**Response:** We think this will add to the complexity of the CONSORT diagram and will make the diagram difficult for the reader to interpret easily.

**Reviewer's question : **Add a brief explanation of the GIFT-CF 1 study (overall aim, methods and perhaps findings)

**Response: **This is a separate study and is cited as reference 13. 

 Ng C, Dellschaft NS, Hoad CL, et al.: Postprandial changes in gastrointestinal function and transit in cystic fibrosis assessed by Magnetic Resonance Imaging. J Cyst Fibros. 2020; 20(4): 591–7. 

https://doi.org/10.1016/j.jcf.2020.06.004

**Reviewer's question : **Please clarify if higher scores of the various patient-reported outcomes indicate worse or better outcomes.  

**Response: **For the PAC-SYM, CFAbdScore and the Likert score, a high score = more severe symptoms.

**Reviewer's question :**

 Discussion: Please comment on potential impact of the double-blinding vs observer-blinding. Do you consider your outcomes to be prone to bias when the participant is not blinded? I would assume that your primary outcome is objective enough to not be affected by detection bias, so why have you chosen the smaller blinded group for your primary analysis?

**Response: **Thank you for raising this important point. We agree that the MRI studies are an objective measure and are unlikely to be subject to detection bias - particularly as the staff conducting and interpreting the scans were blinded.

 The primary analysis was specified in our clinical trials.gov protocol which we published before commencing this trial and amended in the light of the COVID pandemic. The COVID amendment says: "Our primary analysis will be on the patients who completed the trial per protocol but we will present a secondary analysis on all 12 patients, comparing outcome measures on Symkevi ^TM^ with their outcome measures previously recorded at baseline (off treatment)..."

 See: https://clinicaltrials.gov/study/NCT04006873?tab=table 

 This aligns with the METHODS section, under "Statistical analysis", we state: "In view of COVID-19 disruptions, we completed a primary per protocol analysis for all participants who were blind to treatment. A secondary post-hoc intention-to-treat analysis compared all enrolled participants who attended study visits on and off TEZ/IVA.".

# Reviewer report: Reviewer response for version 2

**DOI:** 10.3310/nihropenres.14726.r31310

**Published:** 2024-03-29

**Authors:** Onur Cil

**Affiliations:** 1 University of California San Diego, San Diego, California, USA

My concerns are addressed.

Is the work clearly and accurately presented and does it cite the current literature?

Yes

If applicable, is the statistical analysis and its interpretation appropriate?

Yes

Are all the source data underlying the results available to ensure full reproducibility?

No

Is the study design appropriate and is the work technically sound?

Yes

Are the conclusions drawn adequately supported by the results?

Yes

Are sufficient details of methods and analysis provided to allow replication by others?

Yes

Reviewer Expertise:

Gastrointestinal complications of CF,  intestinal physiology

I confirm that I have read this submission and believe that I have an appropriate level of expertise to confirm that it is of an acceptable scientific standard.

# Reviewer report: Reviewer response for version 2

**DOI:** 10.3310/nihropenres.14726.r31309

**Published:** 2024-03-29

**Authors:** Drucy Borowitz

**Affiliations:** 1 University at Buffalo, Buffalo, USA

The authors have made improvements to this manuscript.

Is the work clearly and accurately presented and does it cite the current literature?

Yes

If applicable, is the statistical analysis and its interpretation appropriate?

I cannot comment. A qualified statistician is required.

Are all the source data underlying the results available to ensure full reproducibility?

No

Is the study design appropriate and is the work technically sound?

Yes

Are the conclusions drawn adequately supported by the results?

Yes

Are sufficient details of methods and analysis provided to allow replication by others?

Yes

Reviewer Expertise:

I have conducted CF GI-focused research, including some on GI tract motility.  I do not have any experience with MRI as a method to assess GI function.

I confirm that I have read this submission and believe that I have an appropriate level of expertise to confirm that it is of an acceptable scientific standard.

# Reviewer report: Reviewer response for version 1

**DOI:** 10.3310/nihropenres.14661.r30972

**Published:** 2024-01-04

**Authors:** Drucy Borowitz

**Affiliations:** 1 University at Buffalo, Buffalo, USA

The authors deserve kudos for 1) continuing to explore a novel, objective measure of GI function and 2) persisting with their study despite the COVID-19 epidemic.  Although they are presenting negative data, it adds to our understanding of GI function in people with CF and, as they  note, the opportunity to explore outcomes prior to use of CFTR modulators is likely to be very limited in the future.  The mention in the Discussion of differences in some of the values presented compared to previous MRI studies using healthy volunteers are thought-provoking.

 Title:   Because the authors state, “There are insufficient data to provide an informative power calculation…” using the word “pilot” in the tile might be appropriate.

 Introduction:

1. A minor point, but in a manuscript focused on GI aspects of CF, saying that it is an epithelial chloride channel rather than an ion channel (or a chloride and bicarbonate channel) seems to ignore the pathophysiology, especially since the role of bicarbonate is mentioned in the Discussion.
2. The introductory statement “In this study, we used magnetic resonance imaging (MRI) ^13^ to evaluate the GI effects…” cites their previous work, but for readers who are unfamiliar with the outcomes of interest, a sentence describing them would be useful.

Methods:

1. Study Procedures:  A clear statement that no participants were taking TEZ-IVA would be better than the explanation that “TEZ/IVA was licensed in the UK but not reimbursed, so was not prescribed in routine clinical care”.

Results:

1. Fecal elastase was measured in all subjects.  It would be good to include the baseline information in Table 3 (Demographics) to indicate whether all subjects were pancreatic insufficient.
2. Figure 7 should not be labeled “Primary analysis”.  I think you mean to say “intent-to-treat analysis”.
3. Values for fecal elastase and calprotectin values should be published in supplemental materials.

Discussion

1. Their primary outcome, OCTT, was notably the same whether subjects were on placebo or TEX/IVA.  They note that other techniques and have found this to be true as well. Do the authors think that, now that they have verified their own prior findings of prolonged OCTT by MRI, this may be a key factor in their findings of high scores for “disorders of bowel movement” on the CF-abd scores? They mention their focus on the IC valve below, but it took a few readings for me to connect these two thoughts.
2. One of the biggest challenges of this study is that it is unclear what the natural variation is in MRI-GI measures in pwCF.  OCTT seems fairly consistent over time, whether subjects were on TEZ/IVA or not.  However, some of the secondary outcomes where individual data is presented indicate that values may vary based on biology as well as on intervention. For example, in Figure 6, SBW increased in 6 subjects and was either stable or decreased in 6 subjects.  Can you attribute that to natural variation?
3. This paragraph seems extraneous: “Recent studies ^8, 9^ have suggested pancreatic exocrine function recovery with ivacaftor after 24 weeks in younger cohorts… This suggests that, if pancreatic recovery were possible, it may occur only in the younger population or require a longer treatment period with CFTR modulators or more efficacious CFTR modulators.” The authors don’t report on feal elastase data other than to say it was unchanged.  The literature suggests that older patients, such as the subjects in this study, may not have changes in pancreatic function.  I think this paragraph should be deleted.
4. The paragraph stating that the authors saw no significant difference in GI symptoms after TEZ/IVA should be combined with the paragraph that follows it, which offers additional explanations for this observation.
5. The large variation between individuals (e.g. large error bars in Figure 5) indicates that GI motility is highly variable.  This should be included as one of the limitations of this study.

Is the work clearly and accurately presented and does it cite the current literature?

Yes

If applicable, is the statistical analysis and its interpretation appropriate?

I cannot comment. A qualified statistician is required.

Are all the source data underlying the results available to ensure full reproducibility?

No

Is the study design appropriate and is the work technically sound?

Yes

Are the conclusions drawn adequately supported by the results?

Yes

Are sufficient details of methods and analysis provided to allow replication by others?

Yes

Reviewer Expertise:

I have conducted CF GI-focused research, including some on GI tract motility.  I do not have any experience with MRI as a method to assess GI function.

I confirm that I have read this submission and believe that I have an appropriate level of expertise to confirm that it is of an acceptable scientific standard.

# Response

**Published:** 2024-02-18

**Authors:** Alan Smyth

**Affiliations:** Lifespan and population health, University of Nottingham, Nottingham, England, UK

**Response to reviewer comments**

 Thank you to our reviewers for taking the time to peer review our manuscript. A detailed response to your comments is outlined below.

**Peer reviewer feedback 1: Drucy Borowitz**

**Title**

 Because the authors state, “There are insufficient data to provide an informative power calculation…” using the word “pilot” in the tile might be appropriate.

**Response: **Added *“a pilot study”* to the title

**Introduction**

**Reviewer comment: **A minor point, but in a manuscript focused on GI aspects of CF, saying that it is an epithelial chloride channel rather than an ion channel (or a chloride and bicarbonate channel) seems to ignore the pathophysiology, especially since the role of bicarbonate is mentioned in the Discussion.

**Response: **Amended to: *“an epithelial ion channel”*

**Reviewer comment: **The introductory statement “In this study, we used magnetic resonance imaging (MRI) ^13^ to evaluate the GI effects…” cites their previous work, but for readers who are unfamiliar with the outcomes of interest, a sentence describing them would be useful.

**Response: **Amended sentence to read:

*“In this study, we used a 6 hour series of magnetic resonance imaging (MRI) ^13^ scans to evaluate the effects of a dual combination CFTR modulator on orocaecal transit time, colon volumes, small bowel water content and their drop in response to a high-calorie meal, and gastric emptying in CF. We also studied the relationship of MRI findings to GI symptoms.”*

**Methods**

**Reviewer comment: **Study Procedures:  A clear statement that no participants were taking TEZ-IVA would be better than the explanation that “TEZ/IVA was licensed in the UK but not reimbursed, so was not prescribed in routine clinical care”.

**Response: **Added sentence:

*“Participants were excluded from the study if they were taking any CFTR modulators.”*

**Results**

**Reviewer comment: **Fecal elastase was measured in all subjects.  It would be good to include the baseline information in Table 3 (Demographics) to indicate whether all subjects were pancreatic insufficient.

**Response: **Faecal elastase and faecal calprotectin now included in Table 3.

**Reviewer comment: **Figure 7 should not be labeled “Primary analysis”.  I think you mean to say “intent-to-treat analysis”.

**Response: **This was the per protocol analysis and therefore have amended the label

**Reviewer comment: **Values for fecal elastase and calprotectin values should be published in supplemental materials.

**Response: **There is no supplementary file, therefore the faecal elastase and calprotectin values have remained in Table 4.

**Discussion**

**Reviewer comment: **Their primary outcome, OCTT, was notably the same whether subjects were on placebo or TEX/IVA.  They note that other techniques have found this to be true as well. Do the authors think that, now that they have verified their own prior findings of prolonged OCTT by MRI, this may be a key factor in their findings of high scores for “disorders of bowel movement” on the CF-abd scores? They mention their focus on the IC valve below, but it took a few readings for me to connect these two thoughts.

**Response: **Thank you for your comment. It is possible that a high CFAbd-score for the “disorders of bowel movement” domain could be due to prolonged OCTT, however it is likely that other factors also contribute. In the 6 ^th^ paragraph of the discussion we now say:

*“However, the GI abnormalities observed using MRI, may contribute to the high scores in disorders of bowel movement.”*

**Reviewer comment: **One of the biggest challenges of this study is that it is unclear what the natural variation is in MRI-GI measures in pwCF.  OCTT seems fairly consistent over time, whether subjects were on TEZ/IVA or not.  However, some of the secondary outcomes where individual data is presented indicate that values may vary based on biology as well as on intervention. For example, in Figure 6, SBW increased in 6 subjects and was either stable or decreased in 6 subjects.  Can you attribute that to natural variation?

**Response: **Figure 6 represents the change in SBW (delta SBW) after the second meal. This is a new metric that we have measured and therefore it is possible that the delta SBW may be more variable in CF than in healthy controls.

 Since there are limited data on this metric, it is difficult to conclude whether this is a normal variation or due to sample size.

**Reviewer comment: **This paragraph seems extraneous: “Recent studies8,9 have suggested pancreatic exocrine function recovery with ivacaftor after 24 weeks in younger cohorts… This suggests that, if pancreatic recovery were possible, it may occur only in the younger population or require a longer treatment period with CFTR modulators or more efficacious CFTR modulators.” The authors don’t report on fecal elastase data other than to say it was unchanged.  The literature suggests that older patients, such as the subjects in this study, may not have changes in pancreatic function.  I think this paragraph should be deleted.

**Response: **Thank you for your comment, this paragraph has been deleted

**Reviewer comment: **The paragraph stating that the authors saw no significant difference in GI symptoms after TEZ/IVA should be combined with the paragraph that follows it, which offers additional explanations for this observation.

**Response: **We have now linked the 2 paragraphs.

**Reviewer comment: **The large variation between individuals (e.g. large error bars in Figure 5) indicates that GI motility is highly variable.  This should be included as one of the limitations of this study.

**Response: **Thank you for your comment. We have acknowledged the variability in the discussion strengths and weaknesses.

**Peer reviewer feedback 2: Onur Cil**

**Reviewer comment: **OCTT primarily accounts for gastric emptying and small intestinal transit. However, majority of gut transit time is actually spent in colon, which is a more relevant intestinal segment for commonly seen constipation in CF. The limitations of OCTT for assessing gut transit and its relevance to CF need to be discussed in the paper

**Response: **Thank you for your comment. We agree that there are limitations in only assessing OCTT and there is limited evidence on colonic or whole gut transit. Previous studies have demonstrated differences in small bowel transit only and not a significant difference in colonic or whole gut transit. Therefore we have added the following sentences:

*“However, it has been previously reported that there is no difference in colonic and whole gut transit times in CF compared to controls ^25^ or in CF patients at baseline compared to measurements during ivacaftor treatment ^11^. It may be beneficial to assess these colonic and whole gut transit times, however, it would add to the participant burden and study costs.”*

**Results**

**Reviewer comment: **What was the average (or median) interval between the current study and baseline scan in the GIFT-CF1 study for 7 patients?

**Response: **Thank you for your question. The median was 45 weeks. We have now included this in the results - study progress section.

**Reviewer comment: **Do authors expect any differences in MRI characteristics over several months/years in the same subject assuming these two studies were done at different times?

**Response: **The MRI values for our outcome measures in the present study are similar to data from our previous studies and therefore we can assume that any differences will be within the normal variation. The comparisons made were within-participant avoiding any between individual variation.

 There are no data to our knowledge which compare MRI characteristics over several months/years in healthy controls or CF. However, another reason for using previous scan data was to reduce the study burden for participants.

**Reviewer comment: **Is there any literature evidence for consistency of MRI findings between measurements done at different time points in people with or without CF?

**Response: **There have been several studies by our group using the same MRI metrics with similar baseline measures in healthy controls. To our knowledge, there are no such data for people with CF.

 One meta-analysis by Dellschaft et al ^1^demonstrated that the small bowel water post-prandial profile is predictable in healthy controls across 29 studies. This included an analysis of the coefficient of variation in n=285 participants taking part in two or more scans, across 20 studies. The coefficient of variation was relatively high at 45%, with a very stable mean SBWC when repeatedly measuring in the same group of participants (mean of 81ml vs. 83ml ie. 2% variability of the mean between two visits).

 In a similar analysis of fasting ascending colon chyme water (T1) in n=40 on 3 separate occasions, the coefficient of variation was 32%, with 8% variability of the mean between 3 visits (0.72s vs. 0.62s vs. 0.68s; currently under review for publication).

**REFERENCES**

 1.         Dellschaft N, Hoad C, Marciani L, Gowland P, Spiller R. Small bowel water content assessed by MRI in health and disease: a collation of single-centre studies. *Aliment Pharmacol Ther* 2022; **55**(3): 327-38.

# Reviewer report: Reviewer response for version 1

**DOI:** 10.3310/nihropenres.14661.r30806

**Published:** 2023-12-14

**Authors:** Onur Cil

**Affiliations:** 1 University of California San Diego, San Diego, California, USA

This randomized, crossover clinical trial investigates effects of CFTR modulator treatment (TEZ/IVA) on gastrointestinal symptoms and abdomen MRI findings (surrogate for gut transit and function) in people with CF. The study is well-designed and results are interpreted reasonably. Although the sample size is small, this is understandable considering that CF is a rare disease. There are a few minor points that need to be addressed prior to publication:

 -OCTT primarily accounts for gastric emptying and small intestinal transit. However, majority of gut transit time is actually spent in colon, which is a more relevant intestinal segment for commonly seen constipation in CF. The limitations of OCTT for assessing gut transit and its relevance to CF need to be discussed in the paper.

 -Results, second paragraph:

1. What was the average (or median) interval between the current study and baseline scan in the GIFT-CF1 study for 7 patients?
2. Do authors expect any differences in MRI characteristics over several months/years in the same subject assuming these two studies were done at different times?
3. Is there any literature evidence for consistency of MRI findings between measurements done at different time points in people with or without CF?

These issues should also be addressed as a potential limitation.

Is the work clearly and accurately presented and does it cite the current literature?

Yes

If applicable, is the statistical analysis and its interpretation appropriate?

Yes

Are all the source data underlying the results available to ensure full reproducibility?

No

Is the study design appropriate and is the work technically sound?

Yes

Are the conclusions drawn adequately supported by the results?

Yes

Are sufficient details of methods and analysis provided to allow replication by others?

Yes

Reviewer Expertise:

Gastrointestinal complications of CF,  intestinal physiology

I confirm that I have read this submission and believe that I have an appropriate level of expertise to confirm that it is of an acceptable scientific standard, however I have significant reservations, as outlined above.
